# Development and utility assessment of a machine learning bloodstream infection classifier in pediatric patients receiving cancer treatments

**DOI:** 10.1186/s12885-020-07618-2

**Published:** 2020-11-13

**Authors:** Lillian Sung, Conor Corbin, Ethan Steinberg, Emily Vettese, Aaron Campigotto, Loreto Lecce, George A. Tomlinson, Nigam Shah

**Affiliations:** 1 Division of Haematology/Oncology, The Hospital for Sick Children, 555 University Avenue, Toronto, Ontario M5G1X8 Canada; 2 Biomedical Informatics Research, Stanford University, Palo Alto, USA; 3 Division of Infectious Diseases, The Hospital for Sick Children, Toronto, Canada; 4 Division of Neonatology, The Hospital for Sick Children, Toronto, Canada; 5 Department of Medicine, University Health Network, Toronto, Canada

**Keywords:** Machine learning, Classifier, Bloodstream infection, Children, Cancer

## Abstract

**Background:**

Objectives were to build a machine learning algorithm to identify bloodstream infection (BSI) among pediatric patients with cancer and hematopoietic stem cell transplantation (HSCT) recipients, and to compare this approach with presence of neutropenia to identify BSI.

**Methods:**

We included patients 0–18 years of age at cancer diagnosis or HSCT between January 2009 and November 2018. Eligible blood cultures were those with no previous blood culture (regardless of result) within 7 days. The primary outcome was BSI. Four machine learning algorithms were used: elastic net, support vector machine and two implementations of gradient boosting machine (GBM and XGBoost). Model training and evaluation were performed using temporally disjoint training (60%), validation (20%) and test (20%) sets. The best model was compared to neutropenia alone in the test set.

**Results:**

Of 11,183 eligible blood cultures, 624 (5.6%) were positive. The best model in the validation set was GBM, which achieved an area-under-the-receiver-operator-curve (AUROC) of 0.74 in the test set. Among the 2236 in the test set, the number of false positives and specificity of GBM vs. neutropenia were 508 vs. 592 and 0.76 vs. 0.72 respectively. Among 139 test set BSIs, six (4.3%) non-neutropenic patients were identified by GBM. All received antibiotics prior to culture result availability.

**Conclusions:**

We developed a machine learning algorithm to classify BSI. GBM achieved an AUROC of 0.74 and identified 4.3% additional true cases in the test set. The machine learning algorithm did not perform substantially better than using presence of neutropenia alone to predict BSI.

**Supplementary Information:**

The online version contains supplementary material available at 10.1186/s12885-020-07618-2.

## Background

Over the last few decades, continued improvement in survival for children with cancer has been observed [1]. Favorable survival outcomes have arisen from better risk stratification, improved understanding of the biology of pediatric cancer and intensification of therapy for some cancer types. Supportive care is also an integral component of cancer management. One of the most important toxicities of cancer treatment is bloodstream infection (BSI), defined as a microbial pathogen isolated from a blood culture. BSIs are important because they are responsible for considerable morbidity, healthcare utilization and treatment-related mortality [2, 3]. More specifically, BSIs may result in infection-related mortality in children who might otherwise be cured [4, 5]. Patients without cancer undergoing hematopoietic stem cell transplantation (HSCT) are also at risk for life-threatening BSI [6]. Identifying the risk of BSI is important as those at lower risk may benefit from less intensive interventions such as outpatient management of fever, while those at higher risk may benefit from more intensive interventions such as broader empiric antibiotics or antibacterial prophylaxis [7, 8].

Even among children receiving identical chemotherapy, the risk of BSI is highly variable [2, 9]. Fever occurring during severe neutropenia (typically defined as an absolute neutrophil count (ANC) less than 0.5 × 10^9^/L) was one of the earliest identified risk factors that predicted life-threatening BSI in patients receiving cancer treatments [10]. While neutropenia is an important risk factor, other factors including other laboratory parameters, bone marrow disease, underlying cancer type, treatments, inpatient status and comorbidities are also thought to be important [11]. While multiple risk stratification schemas have been developed, primarily in the setting of fever and neutropenia (FN), none are universally applicable. More specifically, rules developed in one context may not be valid in a different context [11]. Further, most rules are applied to patients with FN and thus, will miss episodes of BSI occurring in non-neutropenic patients.

The advent of at least two developments may offer both improved and individualized risk prediction for BSI in pediatric patients receiving cancer treatments. First, the transition to electronic health records (EHR) in many pediatric cancer centers in high income countries allows an opportunity to capitalize on these data at little incremental cost [12–14]. Second, machine learning approaches have gained popularity with the introduction of more powerful computing ability combined with development of newer learning algorithms [15]. Together, these developments may permit the creation of new classifiers or machine learning algorithms to detect BSI. If successful, such a classifier could be useful in multiple ways. First, it could supplant using neutropenia as the primary indication to start empiric antibiotics in patients with fever. Second, it could be used in conjunction with neutropenia to identify additional patients who may benefit from empiric antibiotics.

The objectives were to build a machine learning algorithm to identify BSI among children and adolescents with cancer and pediatric HSCT recipients, and to compare this approach with presence of neutropenia to identify BSI.

## Methods

We conducted a retrospective study using data in the EHR (Allscripts) and a separate pediatric cancer database at The Hospital for Sick Children (SickKids), Toronto, Canada. The study was approved by the Research Ethics Board at SickKids (SickKids REB); the need for informed consent was waived given the retrospective nature of the study.

### Eligibility

Eligible patients were 0 to 18 years of age at cancer diagnosis or HSCT (for those without cancer) in whom the diagnosis (or HSCT) occurred between January 1, 2009 and November 6, 2018.

Eligible blood cultures were those in patients without a previous blood culture (regardless of result) within 7 days prior to attempt to capture “new” potentially infectious episodes. This approach also addressed the scenario where multiple cultures were taken and only one was positive, and excluded negative cultures taken after initiation of empiric antibiotics for FN (presuming a culture was obtained prior to initiating antibiotics). We excluded blood cultures obtained prior to 28 days before either cancer diagnosis or HSCT (in those without cancer).

### Procedures

Cancer-specific variables including cancer diagnosis details, relapse date and allogeneic or autologous HSCT dates were obtained from a divisional pediatric cancer database maintained by trained dedicated data managers. Down syndrome status was identified through chart review. Microbiology results and all other features were obtained from the EHR.

### Outcomes

The primary outcome (label) was BSI. BSI was defined as a positive blood culture that was not a contaminant with a common commensal. Common commensals were those delineated by the National Healthcare Safety Network - Centers for Disease Control list of common commensals [16]. The common commensal list was modified to exclude viridans group streptococci given their known association with sepsis syndrome in children and adolescents with cancer [17]. Multiple positive cultures for common commensals were classified as BSI (rather than a contaminant) if two or more positive cultures occurred on the same day or 1 day apart [16].

### Potential predictors (features)

Feature engineering was conducted based upon variables expected to be potentially associated with BSI based on previous research [2, 9, 18–21] and clinical impression. Demographic variables included sex, age, Down syndrome, cancer diagnosis (categorized using the International Classification of Childhood Cancer main category [22]), ordinal cancer diagnosis (for example, primary or secondary cancer), relapse status and previous allogeneic or autologous HSCT at the time the culture was obtained.

The culture location (clinic, emergency department, intensive care unit or hospital ward) was identified. Hospital encounters, pathology tests and radiology tests within 28 days were counted. Hospital encounter features were the number of emergency department visits, number of admissions and total number of encounters including clinic visits. In terms of blood bank utilization, the number of platelet and red blood cell transfusions within the previous 7 days were included. Another feature was the number of prior positive blood cultures in the previous 365 days including common commensals. Antimicrobial exposure within the previous 7 days was also considered. More specifically, administration of systemic antibacterial agents used for the empiric management of FN and systemic antifungal agents were included. Administration one and 2 days prior to the blood culture and the number of days received over the previous 7 days were calculated. These same metrics were calculated for levofloxacin and caspofungin because of their utilization as infection prophylaxis in pediatric patients with cancer [23, 24].

In terms of laboratory values, we evaluated results in the 10,080 min prior to the culture (seven days) and specifically focused on the following: hematology: white blood cell count, ANC (neutrophils plus bands) and neutropenia (ANC less than 0.5 × 10^9^/L); and chemistry: blood urea nitrogen, creatinine, renal failure (creatinine at least 1.5 times upper limit of normal), albumen, alanine aminotransferase, glucose, lactate, pH (arterial, capillary and venous) and sodium. For actual values, four quantities were evaluated: 0 to < 24 h prior, 24 to < 48 h prior, average over the 7 days and either the minimum or the maximum over the 7 days. If the ANC within 24 h of the blood culture was missing, we imputed the ANC from one, two, or 3 days prior in that order. For most laboratory values, the minimum or maximum was chosen based upon the extreme associated with sicker patients. However, since both high and low glucose and sodium can be deleterious, both the maximum and minimum were calculated in these cases. If the same test was performed multiple times within a 24-h period, the value closest to the culture (the later result) was used and thus, only one value per day contributed to 0 to < 24 h prior, 24 to < 48 h prior and average values.

### Analysis

Baseline characteristics were compared between the BSI and non-BSI groups using the Student’s t-test for continuous variables and the chi square test for categorical variables. The data set was divided into training (60%), validation (20%) and test (20%) sets separated sequentially in time to avoid look-ahead bias.

Four machine learning algorithms were used, namely elastic net, support vector machine and two implementations of gradient boosting machine (GBM and XGBoost). These were implemented using the Caret package in R [25]. As a general strategy, the models were trained and the parameters were tuned using the training set. The models were then implemented in the validation set and a single model was chosen to be applied to the test set. Model selection was based upon the area-under-the-receiver-operator-curve (AUROC) and diagnostic test properties (sensitivity, specific, positive predictive value, and negative predictive value). In order to compare models, we a priori decided that a new algorithm would be unacceptable if it resulted in more false negatives (failed to detect BSI) than using neutropenia alone. Thus, we set the diagnostic testing threshold such that the number of false negatives would match the number of false negatives using neutropenia within the previous 24 h. Once a model was selected, the model was re-trained and parameters were re-tuned using the combined training and validation sets (80%). The final model was then applied to the test set.

For data preparation, laboratory features that were missing in more than 80% of eligible blood cultures were removed. Other missing values were imputed singly using chained equations via the MICE package in R [26]. Imputation was performed separately in the training, validation and test sets. Other model pre-processing steps consisted of centering, scaling and removing near-zero variance features. A grid search was used for parameter tuning. Models were trained using five cross-validation folds repeated five times in which the metric monitored was the AUROC. Both in-sample (cross-validation) and out-of-sample AUROCs were reported. All analyses were performed using R studio version 3.6.1, The R Foundation for Statistical Computing.

## Results

There were 11,183 eligible blood cultures from 2306 patients included in the analysis. Overall, the number of positive BSI was 624 (5.6%). Baseline characteristics and features are shown in Tables 1, 2, 3 stratified by BSI. More detailed cancer diagnosis is shown in Additional file 1: Appendix 1; the most common cancer was lymphoid leukemia (4140, 37.0%). Additional file 1: Appendix 2 shows the lab values that were removed because they were missing in over 80% of blood culture episodes; most related to lactate dehydrogenase and arterial, capillary or venous pH values. Table 4 and Additional file 1: Appendix 3 illustrate the isolates associated with BSI. The most common pathogens were coagulase negative staphylococci, viridans group streptococci, *Escherichia species*, *Staphylococcus aureus* and *Pseudomonas aeruginosa* in descending order. Additional file 1: Appendix 4 illustrates the number of eligible blood cultures, number of unique patients, number of positive cultures and number of unique patients with positive cultures within the training (*n* = 6710 cultures), validation (*n* = 2237 cultures) and test (*n* = 2236 cultures) sets separately.

**Table 1:** Demographics of the Cohort Stratified by Bloodstream Infection (*N* = 11,183 cultures)

	Negative	Positive	P*
n	10,559	624	
**Male Sex**	5841 (55.3)	330 (52.9)	0.252
**Mean Age (SD**)	7.10 (4.82)	7.47 (5.11)	0.062
**Down Syndrome**	263 (2.5)	26 (4.2)	0.015
**ICCC Category**			< 0.001
I. Leukemias, Myeloproliferative Diseases and Myelodysplastic Diseases	5070 (48.0)	403 (64.6)	
II. Lymphomas and Reticuloendothelial Neoplasms	1325 (12.5)	56 (9.0)	
III. Central Nervous System Tumors	1223 (11.6)	52 (8.3)	
IV. Neuroblastoma	899 (8.5)	48 (7.7)	
V. Retinoblastoma	127 (1.2)	3 (0.5)	
VI. Renal Tumors	248 (2.3)	2 (0.3)	
VII. Hepatic Tumors	168 (1.6)	4 (0.6)	
VIII. Malignant Bone Tumors	528 (5.0)	15 (2.4)	
IX. Soft-tissue and Other Extraosseous Sarcomas	573 (5.4)	20 (3.2)	
X. Germ Cell Tumors	146 (1.4)	3 (0.5)	
XI. Other Malignant Epithelial Neoplasms	103 (1.0)	6 (1.0)	
XII. Other and unspecified malignant neoplasms	19 (0.2)	1 (0.2)	
Not cancer undergoing HSCT	130 (1.2)	11 (1.8)	
**Ordinal Cancer**			0.904
1	10,446 (98.9)	617 (98.9)	
2	105 (1.0)	7 (1.1)	
3 or 4	8 (0.1)	0 (0.0)	
**Relapse**	1132 (10.7)	134 (21.5)	< 0.001
**Prior Hematopoietic Stem Cell Transplant**			
Allogeneic	739 (7.0)	94 (15.1)	< 0.001
Autologous	914 (8.7)	61 (9.8)	0.373

*Abbreviations*: *SD* Standard deviation, *ICCC* International Classification of Childhood Cancer, *HSCT* Hematopoietic stem cell transplantation

*P values calculated using Student’s t-test for continuous variables and chi square test for categorical variables

**Table 2:** Preceding Healthcare Encounters, Tests, Blood Bank Utilization and Systemic Antibiotic Administration (*N* = 11,183 cultures)

	Negative	Positive	P*
N	10,559	624	
**Location where Culture Obtained**			< 0.001
Clinic	1346 (12.7)	62 (9.9)	
Emergency Department	4877 (46.2)	219 (35.1)	
Intensive Care Unit	329 (3.1)	9 (1.4)	
Hospital Ward	4007 (37.9)	334 (53.5)	
**Healthcare Encounters within 28 Days**			
Mean Number Emergency Department Visits (SD)	0.35 (0.62)	0.28 (0.54)	0.006
Mean Number Admissions (SD)	0.53 (0.71)	0.57 (0.67)	0.178
Mean All Encounters (SD)	3.95 (3.32)	3.86 (3.06)	0.474
**Tests Performed within 28 Days**			
Mean Pathology Specimens (SD)	0.24 (0.77)	0.39 (1.04)	< 0.001
Mean Radiology Tests (SD)	1.12 (2.07)	1.37 (2.14)	0.003
**Blood Bank Utilization within 7 Days**			
Mean Platelet Transfusions (SD)	0.40 (1.20)	1.15 (1.78)	< 0.001
Mean Red Cell Transfusions (SD)	0.28 (0.80)	0.53 (0.91)	< 0.001
**Previous Positive Blood Cultures 7–365 Days**			
Mean Positive Cultures (SD)	0.65 (1.95)	1.18 (2.22)	< 0.001
**Systemic Antibiotics within 7 Days**			
FN Antibiotics One Day Prior (%)	273 (2.6)	11 (1.8)	0.255
FN Antibiotics Two Days Prior (%)	250 (2.4)	14 (2.2)	0.950
Mean Days FN Antibiotics (SD)	0.24 (0.86)	0.35 (0.94)	0.001
Antifungal One Day Prior (%)	1008 (9.5)	210 (33.7)	< 0.001
Antifungal Two Days Prior (%)	936 (8.9)	208 (33.3)	< 0.001
Mean Days Antifungal (SD)	0.56 (1.56)	1.94 (2.56)	< 0.001
Levofloxacin One Day Prior (%)	36 (0.3)	8 (1.3)	0.001
Levofloxacin Two Days Prior (%)	45 (0.4)	5 (0.8)	0.291
Mean Days Levofloxacin (SD)	0.02 (0.25)	0.08 (0.50)	< 0.001
Caspofungin One Day Prior (%)	380 (3.6)	55 (8.8)	< 0.001
Caspofungin Two Days Prior (%)	349 (3.3)	56 (9.0)	< 0.001
Mean Days Caspofungin (SD)	0.22 (1.01)	0.56 (1.58)	< 0.001

*Abbreviations*: *SD* Standard deviation, *FN* Fever and neutropenia

*P values calculated using Student’s t-test for continuous variables and chi square test for categorical variables

**Table 3:** Preceding Laboratory Values within Seven Days (*N* = 11,183 cultures)^a^

	Negative	Positive	P***
n	10,559	624	
**WBC, ANC and Neutropenia**			
Mean WBC 0- < 24 Hours (SD)	8.39 (27.04)	4.38 (13.54)	< 0.001
Mean WBC 24- < 48 Hours (SD)	5.52 (12.33)	1.67 (6.80)	< 0.001
Mean Average WBC Prior 7 Days (SD)	8.24 (26.29)	4.70 (13.31)	0.001
Mean Minimum WBC Prior 7 Days (SD)	6.90 (26.08)	3.73 (13.03)	0.003
Mean ANC 0- < 24 Hours (SD)	4.15 (6.50)	2.66 (4.73)	< 0.001
Mean ANC 24- < 48 Hours (SD)	3.38 (6.32)	1.10 (3.21)	< 0.001
Mean Average ANC Prior 7 Days (SD)	3.96 (5.91)	2.73 (4.15)	< 0.001
Mean Minimum ANC Prior 7 Days (SD)	3.00 (5.37)	1.90 (3.61)	< 0.001
Number Neutropenic 0- < 24 Hours (%)^b^	2745 (26.0)	397 (63.6)	< 0.001
Number Neutropenic 24- < 48 Hours (%)^b^	1146 (10.9)	253 (40.5)	< 0.001
**Renal Function**			
Mean BUN 0- < 24 Hours (SD)	4.41 (2.79)	4.88 (2.85)	0.001
Mean BUN 24- < 48 Hours (SD)	4.65 (3.34)	5.22 (3.04)	0.011
Mean Average BUN Prior 7 Days (SD)	4.47 (2.60)	4.86 (2.41)	0.001
Mean Maximum BUN Prior 7 Days (SD)	5.07 (3.20)	5.66 (3.01)	< 0.001
Mean Creatinine 0- < 24 Hours (SD)	37.35 (35.55)	34.16 (14.24)	0.039
Mean Creatinine 24- < 48 Hours (SD)	36.32 (39.38)	34.25 (14.47)	0.367
Mean Average Creatinine Prior 7 Days (SD)	36.93 (35.50)	34.18 (13.66)	0.062
Mean Maximum Creatinine Prior 7 Days (SD)	39.67 (40.39)	37.93 (16.18)	0.299
Renal Failure 0- < 24 Hours (%)^b^	122 (1.2)	2 (0.3)	0.082
Renal Failure 24- < 48 Hours (%)^b^	48 (0.5)	1 (0.2)	0.441
Renal Failure Prior 7 Days (%)	164 (1.6)	5 (0.8)	0.184
**Other Laboratory Values**			
Mean Albumen 0- < 24 Hours (SD)	35.02 (6.33)	34.22 (5.99)	0.102
Mean Average Albumen Prior 7 Days (SD)	35.44 (6.13)	34.57 (5.36)	0.012
Mean Minimum Albumen Prior 7 Days (SD)	34.08 (6.64)	33.01 (5.82)	0.005
Mean ALT 0- < 24 Hours (SD)	81.28 (159.48)	78.32 (79.34)	0.785
Mean Average ALT Prior 7 Days (SD)	77.01 (123.52)	78.70 (89.95)	0.781
Mean Maximum Prior 7 Days ALT (SD)	87.07 (146.59)	92.97 (116.53)	0.415
Mean Glucose 0- < 24 Hours (SD)	5.59 (2.31)	5.60 (1.83)	0.928
Mean Glucose 24- < 48 Hours (SD)	5.51 (2.04)	5.64 (2.32)	0.310
Mean Average Glucose Prior 7 Days (SD)	5.54 (1.64)	5.61 (1.40)	0.341
Mean Minimum Glucose Prior 7 Days (SD)	5.09 (1.45)	4.93 (1.07)	0.013
Mean Maximum Glucose Prior 7 Days (SD)	6.17 (3.01)	6.55 (2.79)	0.004
Mean Sodium 0- < 24 Hours (SD)	138.22 (3.34)	137.54 (3.86)	< 0.001
Mean Sodium 24- < 48 Hours (SD)	139.13 (3.38)	138.97 (3.27)	0.434
Mean Average Sodium Prior 7 Days (SD)	138.62 (2.96)	138.27 (3.28)	0.006
Mean Minimum Sodium Prior 7 Days (SD)	137.59 (3.20)	136.65 (3.68)	< 0.001
Mean Maximum Sodium Prior 7 Days (SD)	139.65 (3.46)	139.82 (3.77)	0.255

*Abbreviations*: *SD* Standard deviation, *ANC* Absolute neutrophil count, *WBC* White blood cell count, *BUN* Blood urea nitrogen, *ALT* Alanine aminotransferase

^a^ Units: WBC 10^9^/L, ANC 10^9^/L, BUN mmol/L, creatinine umol/L, albumen g/L, ALT U/L, glucose mmol/L, sodium mmol/L

^b^ Neutropenia defined as ANC < 0.5 × 10^9^/L; renal failure defined as serum creatinine ≥1.5 times upper limit of normal

*** *P* values calculated using Student’s t-test for continuous variables and chi square test for categorical variables

**Table 4:** Most Common Bloodstream Infection Microorganisms

Species	Frequency
Coagulase Negative Staphylococci	148
Viridans Group Streptococci	140
*Escherichia* species	76
*Staphylococcus aureus*	65
*Pseudomonas aeruginosa*	44
*Enterobacter* species	40
*Klebsiella* species	40
*Micrococcus* species	23
*Streptococcus pneumoniae*	29
*Bacillus* species	21

Additional file 1: Appendix 5 illustrates model performance in the training and validation sets. The in-sample cross-validation AUROC ranged from 0.71 to 0.79 across the four models with XGBoost having the highest cross-validation AUROC of 0.79. In the validation set, the out-of-sample AUROC was 0.77 for elastic net, GBM and XGBoost. Thus, model choice relied upon diagnostic testing properties. The number of false negatives in the validation set with the neutropenia model was 47 of 2237 cultures. Setting the same number of false negatives across all four models, accuracy (fraction of predictions that were correct) was highest for GBM (0.74) and lowest for support vector machine (0.48). In evaluating kappa, sensitivity, specificity, positive predictive value and negative predictive value, GBM was the same or better than the other approaches and thus, the GBM model was chosen. When the model was re-trained and parameters were re-tuned using GBM in the training and validation sets combined, the in-sample AUROC from cross-validation was 0.78. Additional file 1: Appendix 6 illustrates the 20 most important features contributing to the final model. Neutropenia within 24 h was not included in this list.

Table 5 shows the performance of GBM in the test set and compares it to the neutropenia model using the threshold derived when setting the number of false negatives to be the same as in the neutropenia model (52 of 2236 cultures). Specificity was 0.76 with GBM compared to 0.72 with the neutropenia model, resulting in 508 false positives with GBM and 592 with the neutropenia model (difference of 84 cases). Among the 139 with BSI in the test set, 81 were positive by both GBM and neutropenia, 46 were negative by both and six were missed by each model. Among the 2097 with negative BSI, 1356 were negative by both GBM and neutropenia, 359 were positive by both, 149 were false positives with GBM and 233 were false positives with neutropenia. The AUROC of GBM in the test set was 0.74.

**Table 5:** Performance in Test Set (*N* = 2236) ^a^

	Proportion Positive	Sensitivity	Specificity	Positive Predictive Value	Negative Predictive Value	True Positive	False Positive	True Negative	False Negative
GBM^b^	595 (26.6%)	0.63	0.76	0.15	0.97	87	508	1589	52
Neutropenia	679 (30.4%)	0.63	0.72	0.13	0.97	87	592	1505	52

*Abbreviation*: *GBM* one implementation of gradient boosting machine

^a^ Observed bloodstream infection rate 139/2236 (6.2%) in the test set

^b^ Test threshold chosen (probability> 0. 0489) to keep the number of false negatives the same as in the absolute neutrophil count < 0.5 × 10^9^/L model (52/2236)

If GBM was applied in addition to neutropenia as the criteria to initiate empiric antibacterial agents, six non-neutropenic patients would be identified, representing 6/139 (4.3%) of those with BSI. The microorganisms were as follows: coagulase negative staphylococci (*n* = 1), *Acinetobacter spp.* (*n* = 1), *Enterobacter spp*. (*n* = 1), *Proteus spp.* (*n* = 1), *Moraxella spp.* (*n* = 1) and non-albicans *Candida*. Five patients had high-risk FN features (had they been neutropenic) as follows: post allogeneic HSCT (*n* = 3), post autologous HSCT (*n* = 1) and induction chemotherapy for acute lymphoblastic leukemia (*n* = 1). All of these patients with high-risk features received parenteral antibiotics prior to the culture results being known while the sixth patient received oral antibiotics prior to culture result availability. Two experienced bacteria-related sepsis although none died during the episode.

## Discussion

We were successful in using an institutional EHR to develop a machine learning algorithm to predict BSI. While the in-sample and the out-of-sample AUROC were reasonable, the final model did not perform substantially better than the presence of neutropenia at fever onset. Even though the model was able to identify additional BSI in non-neutropenic patients, these predictions would not have provided additional value because all of these patients had received empiric antibiotics. Provision of empiric antibiotics in non-neutropenic patients is standard in patients who appear ill or when clinicians apply their own judgement and experience in identifying higher risk patients.

The developed BSI classifier performance does not support its use as a replacement for neutropenia as an indication for empiric therapy. If used as an adjunct to neutropenia, whether the identification of six additional non-neutropenic BSI cases at the cost of 149 more false positive results is worthwhile will depend on at least two major considerations. First is the advantage of identifying non-neutropenic patients with BSI and whether deploying the model would improve clinically meaningful outcomes [27]. To this point, one of the BSI was a yeast and thus, empiric antibacterial agents would not have led to better results. On the other hand, two of these patients developed bacterial sepsis, emphasizing the importance of early antibiotics in this small cohort. In our setting, all non-neutropenic BSI patients received empiric antibiotics prior to culture result availability and thus, the algorithm would not be useful at our institution. However, such an algorithm could be useful in settings in which limited expertise and resources, or high volumes impede clinicians from deciding which non-neutropenic patients should receive empiric antibacterial agents.

The second consideration is the cost and effort to implement a machine learning algorithm into the clinical workflow of busy clinicians and commonly over-burdened information technology hospital staff. Indeed, for a machine learning model to be successful, it would need to be integrated at any location in which pediatric patients receiving cancer treatments are assessed for fever including clinics, the emergency department and the inpatient ward. This complexity increases the hidden deployment cost [28]. Unless the benefit of a machine learning algorithm is clearly evident, such implementation is likely to be challenging.

There are several potential reasons why the developed algorithm did not perform substantially better than neutropenia alone. First, it is possible that the sample size was too small, particularly when considering the number of unique patients in this data set. Second, it is possible that features most important to predicting BSI were not present in this data set. A notable absence is the lack of flow chart data, which only was implemented into the EHR during the latter part of the study period and thus, could not be used in model building. Future work should focus on identifying other data that could inform a BSI classifier. In comparing classifiers and the best classifier against the neutropenia model, we chose to fix the sensitivity to ensure a new model could not miss more cases of bacteremia. Alternatively, we could have chosen to fix the specificity to see whether the model might have enhanced sensitivity. However, this analysis could also show worse sensitivity, a scenario that would not be considered clinically acceptable.

A strength of this study was the use of a well-curated cancer diagnosis and treatment data set combined with EHR data to build a BSI classifier. A second strength is that it complements the large literature of risk prediction models in FN by expanding the target to all pediatric cancer patients at risk for BSI. However, this study has several limitations. The analysis did not account for the correlated structure of the data in that the same patient could contribute multiple episodes although this may be less problematic since our focus was on prediction rather than inference. Second, different blood draws from the same patient could have been in the training, validation and test sets and this approach may have resulted in overly optimistic results. However, this aspect is likely less problematic as it mimics how the algorithm would be deployed in clinical practice. Finally, our data set was relatively small, and it is possible that the algorithms would have performed better had more data been available.

## Conclusion

In conclusion, we developed a machine learning algorithm to classify BSI. GBM achieved an AUROC of 0.74 and identified 4.3% additional true cases in the test set. The machine learning algorithm did not perform substantially better than using the presence of neutropenia alone to predict BSI.

## Supplementary Information

**Additional file 1.**

## Data Availability

As the data include personal health information, the data are not available publicly.

## Abbreviations

ANC: Absolute neutrophil count
AUROC: Area-under-the-receiver-operator-curve
BSI: Bloodstream infection
EHR: Electronic health records
FN: Fever and neutropenia
HSCT: Hematopoietic stem cell transplantation
SickKids REB: Research Ethics Board at SickKids
SickKids: Hospital for Sick Children
