# Supplementary material for: Development and utility assessment of a machine learning bloodstream infection classifier in pediatric patients receiving cancer treatments
Source: BMC Cancer. 2020 Nov 13;20:1103. doi: 10.1186/s12885-020-07618-2 (PMC7666525; doi:10.1186/s12885-020-07618-2)
Supplement: Supplementary file 1 — Additional file 1. [file 12885_2020_7618_MOESM1_ESM.docx]

**Appendix 1: Detailed ICCC Classification by Bloodstream Infection**

|  | **Negative** | **Positive** | **p** |
| --- | --- | --- | --- |
| n | 10559 | 624 |  |
|  |  |  | <0.001 |
| I. Leukemias, Myeloproliferative Diseases and Myelodysplastic Diseases | 5070 (48.0) | 403 (64.6) |  |
| 1. Lymphoid leukemias | 3924 (37.2) | 216 (34.6) |  |
| 1. Acute myelogenous leukemias | 881 (8.3) | 161 (25.8) |  |
| 1. Chronic myeloproliferative diseases | 27 (0.3) | 3 (0.5) |  |
| 1. Myelodysplastic syndrome | 197 (1.9) | 16 (2.6) |  |
| 1. Unspecified | 41 (0.4) | 7 (1.1) |  |
| II. Lymphomas and Reticuloendothelial Neoplasms | 1325 (12.5) | 56 (9.0) |  |
| 1. Hodgkin lymphomas | 259 (2.5) | 1 (0.2) |  |
| 1. Non-Hodgkin lymphomas (except Burkitt) | 792 (7.5) | 42 (6.7) |  |
| 1. Burkitt lymphoma | 121 (1.1) | 3 (0.5) |  |
| 1. Miscellaneous lymphoreticular | 148 (1.4) | 10 (1.6) |  |
| 1. Unspecified lymphomas | 5 (0.0) | 0 (0.0) |  |
| III. Central Nervous System Tumors | 1223 (116.6) | 52 (8.3) |  |
| 1. Ependymomas and choroid plexus tumors | 108 (1.0) | 5 (0.8) |  |
| 1. Astrocytomas | 266 (2.5) | 6 (1.0) |  |
| 1. Intracranial and intraspinal embryonal | 639 (6.1) | 33 (5.3) |  |
| 1. Other gliomas | 60 (0.6) | 1 (0.2) |  |
| 1. Other specified | 135 (1.3) | 7 (1.1) |  |
| 1. Unspecified | 15 (0.1) | 0 (0.0) |  |
| IV. Neuroblastoma | 899 (8.5) | 48 (7.7) |  |
| 1. Neuroblastoma and ganglioneuroblastoma | 890 (8.4) | 47 (7.5) |  |
| 1. Other peripheral nervous cell tumors | 9 (0.1) | 1 (0.2) |  |
| V. Retinoblastoma | 127 (1.2) | 3 (0.5) |  |
| VI. Renal Tumors | 248 (2.3) | 2 (0.3) |  |
| 1. Nephroblastoma | 232 (2.2) | 2 (0.3) |  |
| 1. Renal carcinomas | 14 (0.1) | 0 (0.0) |  |
| 1. Unspecified malignant renal tumors | 2 (0.0) | 0 (0.0) |  |
| VII. Hepatic Tumors | 168 (1.6) | 4 (0.6) |  |
| 1. Hepatoblastoma | 159 (1.5) | 3 (0.5) |  |
| 1. Hepatic carcinomas | 9 (0.1) | 1 (0.2) |  |
| VIII. Malignant Bone Tumors | 528 (5.0) | 15 (2.4) |  |
| 1. Osteosarcomas | 309 (2.9) | 8 (1.3) |  |
| 1. Ewing tumor | 197 (1.9) | 5 (0.8) |  |
| 1. Other specified malignant bone tumors | 14 (0.1) | 0 (0.0) |  |
| 1. Unspecified malignant bone tumors | 8 (0.1) | 2 (0.3) |  |
| IX. Soft-tissue and Other Extraosseous Sarcomas | 573 (5.4) | 20 (3.2) |  |
| 1. Rhabdomyosarcomas | 304 (2.9) | 11 (1.8) |  |
| 1. Fibrosarcomas and other fibrous neoplasms | 79 (0.7) | 3 (0.5) |  |
| 1. Skin carcinomas | 81 (0.8) | 2 (0.3) |  |
| 1. Other and unspecified carcinomas | 109 (1.0) | 4 (0.6) |  |
| X. Germ Cell Tumors | 146 (1.4) | 3 (0.5) |  |
| 1. Intracranial and intraspinal germ cell tumors | 47 (0.4) | 1 (0.2) |  |
| 1. Malignant extracranial and extragonadal | 42 (0.4) | 2 (0.3) |  |
| 1. Malignant gonadal germ cell tumours | 40 (0.4) | 0 (0.0) |  |
| 1. Gonadal carcinomas | 9 (0.1) | 0 (0.0) |  |
| 1. Other and unspecified malignant gonadal | 8 (0.1) | 0 (0.0) |  |
| XI. Other Malignant Epithelial Neoplasms | 103 (1.0) | 6 (1.0) |  |
| 1. Adrenocortical carcinomas | 35 (0.3) | 2 (0.3) |  |
| 1. Thyroid carcinomas | 8 (0.1) | 0 (0.0) |  |
| 1. Nasopharyngeal carcinomas | 17 (0.2) | 0 (0.0) |  |
| 1. Malignant melanomas | 5 (0.0) | 0 (0.0) |  |
| 1. Skin carcinomas | 1 (0.0) | 0 (0.0) |  |
| 1. Other and unspecified carcinomas | 37 (0.4) | 4 (0.6) |  |
| XII. Other and unspecified malignant neoplasms | 19 (0.2) | 1 (0.2) |  |
| 1. Other specified malignant tumors | 13 (0.1) | 1 (0.2) |  |
| 1. Other unspecified malignant tumors | 6 (0.1) | 0 (0.0) |  |
| Not cancer undergoing HSCT | 130 (1.2) | 11 (1.8) |  |

Abbreviations: ICCC – International Classification of Childhood Cancer; HSCT – hematopoietic stem cell transplantation

**Appendix 2: Laboratory Values Removed as Features Missing > 80% *^,^****

|  | **Negative** | **Positive** | **P** |  | **Missing** |  |
| --- | --- | --- | --- | --- | --- | --- |
|  | n=10559 | n=624 |  |  | n | % |
| Mean Albumen 24-<48 Hours (SD) | 34.70 (5.96) | 34.03 (5.74) | 0.215 |  | 9532 | 85.2 |
| Mean ALT 24-<48 Hours (SD) | 81.25 (126.57) | 88.15 (84.19) | 0.543 |  | 9423 | 84.3 |
| Mean Lactate 0-<24 Hours (SD) | 1.83 (1.42) | 1.73 (0.89) | 0.680 |  | 10552 | 94.4 |
| Mean Lactate 24-<48 Hours (SD) | 1.79 (1.13) | 1.12 (0.43) | 0.192 |  | 11016 | 98.5 |
| Mean Average Lactate (SD) | 1.86 (1.36) | 1.72 (0.89) | 0.533 |  | 10382 | 92.8 |
| Mean Maximum Lactate (SD) | 2.02 (1.46) | 1.79 (0.92) | 0.327 |  | 10382 | 92.8 |
| Mean LDH 0-<24 Hours (SD) | 2188.11 (4314.25) | 1512.35 (929.47) | 0.453 |  | 10331 | 92.4 |
| Mean LDH 24-<48 Hours (SD) | 1388.61 (1582.56) | 911.79 (694.09) | 0.266 |  | 10993 | 98.3 |
| Mean Average LDH (SD) | 1830.87 (3547.08) | 1155.68 (960.03) | 0.163 |  | 9765 | 87.3 |
| Mean Maximum LDH (SD) | 1850.28 (3553.40) | 1175.50 (972.49) | 0.164 |  | 9765 | 87.3 |
| Mean Arterial pH 0-<24 Hours (SD) | 7.35 (0.08) | 7.45 (0.06) | 0.090 |  | 10947 | 97.9 |
| Mean Arterial pH 24-<48 Hours (SD) | 7.36 (0.07) | 7.44 (0.04) | 0.153 |  | 11049 | 98.8 |
| Mean Average Arterial pH (SD) | 7.35 (0.07) | 7.41 (0.05) | 0.153 |  | 10823 | 96.8 |
| Mean Minimum Arterial pH (SD) | 7.34 (0.07) | 7.38 (0.04) | 0.273 |  | 10823 | 96.8 |
| Mean Capillary pH 0-<24 Hours (SD) | 7.36 (0.07) | 7.28 (0.09) | 0.106 |  | 11169 | 99.9 |
| Mean Capillary pH 24-<48 Hours (SD) | 7.34 (0.05) | 7.37 (NC) | NC |  | 11171 | 99.9 |
| Mean Average Capillary pH (SD) | 7.36 (0.04) | 7.31 (0.04) | 0.130 |  | 11154 | 99.7 |
| Mean Minimum Capillary pH (SD) | 7.34 (0.06) | 7.26 (0.08) | 0.029 |  | 11154 | 99.7 |
| Mean Venous pH 0-<24 Hours (SD) | 7.39 (0.07) | 7.41 (0.06) | 0.026 |  | 10441 | 93.4 |
| Mean Venous pH 24-<48 Hours (SD) | 7.36 (0.07) | 7.35 (0.07) | 0.875 |  | 11069 | 99.0 |
| Mean Average Venous pH (SD) | 7.38 (0.07) | 7.40 (0.06) | 0.014 |  | 10183 | 91.1 |
| Mean Minimum Venous pH (D) | 7.38 (0.07) | 7.40 (0.06) | 0.009 |  | 10183 | 91.1 |

Abbreviations: SD – standard deviation; ALT- alanine aminotransferase; LDH – lactate dehydrogenase; NC – not calculatable

* Laboratory features from the 7 days prior to the blood culture

** Units: Albumen g/L, ALT U/L, lactate mmol/L, LDH U/L

**Appendix 3: Microorganisms Identified in Bloodstream Infection**

| **Category** | **Species** | **Frequency** |
| --- | --- | --- |
| **Gram Negative Bacilli** | *Achromobacter* | 1 |
|  | *Acinetobacter* | 9 |
|  | *Burkholderia* | 1 |
|  | *Campylobacter jejuni* | 1 |
|  | *Capnocytophaga species* | 6 |
|  | *Citrobacter species* | 6 |
|  | *Enterobacter species* | 40 |
|  | *Escherichia species* | 76 |
|  | *Haemophilus species* | 5 |
|  | *Klebsiella species* | 40 |
|  | *Leptotrichia species* | 4 |
|  | *Moraxella species* | 12 |
|  | Other Gram negative bacillus | 7 |
|  | *Pantoea species* | 1 |
|  | *Proteus species* | 2 |
|  | *Pseudomonas aeruginosa* | 44 |
|  | *Pseudomonas* non-aeruginosa | 5 |
|  | *Salmonella species* | 1 |
|  | *Serratia marcescens* | 2 |
|  | *Sphingomonas species* | 1 |
|  | *Stenotrophomonas maltophilia* | 8 |
|  | Gram negative bacillus NOS | 2 |
| **Gram Negative Coccus** | *Neisseria species* | 10 |
| **Gram Positive Coccus** | *Enterococcus species* | 11 |
|  | *Micrococcus species* | 23 |
|  | Nutritionally variant streptococci | 4 |
|  | Other Gram positive coccus | 2 |
|  | *Rothia species* | 17 |
|  | *Staphylococcus aureus* | 65 |
|  | *Streptococcus* group ABD | 2 |
|  | *Streptococcus pneumoniae* | 29 |
|  | Viridans group streptococci | 140 |
|  | Coagulase negative staphylococci | 148 |
| **Gram Positive Bacillus** | *Actinomyces* | 2 |
|  | Anaerobic diphtheroid | 1 |
|  | *Bacillus species* | 21 |
|  | *Clostridium species* | 2 |
|  | *Cornynebacterium species* | 9 |
|  | Diphtheroid | 7 |
|  | *Microbacterium species* | 1 |
|  | Other Gram positive bacillus | 2 |
|  | *Propionibacterium species* | 1 |
|  | Gram positive bacillus NOS | 3 |
| **Other** | *Mycobacterium* | 2 |
|  | *Candida albicans* | 3 |
|  | *Candida* non-albicans | 10 |

Abbreviations: NOS- not otherwise specified

**Appendix 4: Number of Cultures and Unique Individuals in Training, Validation and Test**

**Sets**

|  | **Training Set** | **Validation Set** | **Test Set** |
| --- | --- | --- | --- |
| Number Eligible Blood Cultures | 6710 | 2237 | 2236 |
| Number Unique Patients | 1406 | 709 | 692 |
| Number Positive Cultures | 345 | 140 | 139 |
| Number Unique Patients with Positive Cultures | 235 | 107 | 109 |

**Appendix 5: Training and Validation Set Results**

|  | **Elastic Net** | **GBM** | **Support Vector Machine** | **XGBoost** |
| --- | --- | --- | --- | --- |
| **Training Set** |  |  |  |  |
| Median AUROC (in-sample) | 0.76 | 0.78 | 0.71 | 0.79 |
|  |  |  |  |  |
| **Validation Set*** |  |  |  |  |
| AUROC (out-of-sample) | 0.77 | 0.77 | 0.64 | 0.77 |
| **Ideal Threshold*** | 0.0600 | 0.0400 | 0.0355 | 0.0450 |
| Accuracy | 0.72 | 0.74 | 0.48 | 0.72 |
| Kappa | 0.14 | 0.16 | 0.03 | 0.14 |
| Sensitivity (Recall) | 0.66 | 0.66 | 0.66 | 0.66 |
| Specificity | 0.72 | 0.75 | 0.47 | 0.72 |
| PPV (Precision) | 0.14 | 0.15 | 0.08 | 0.14 |
| NPV | 0.97 | 0.97 | 0.95 | 0.97 |
| Prevalence | 0.06 | 0.06 | 0.06 | 0.06 |
| Detection Rate | 0.04 | 0.04 | 0.04 | 0.04 |
| Detection Prevalence | 0.30 | 0.28 | 0.54 | 0.30 |
| Number False Negative | 47 | 47 | 47 | 47 |
|  |  |  |  |  |
| **Train + Validation Set** |  |  |  |  |
| Median AUROC (in-sample) |  | 0.78 |  |  |

Abbreviations: AUROC – area under the receiver operator curve; PPV – positive predictive value; NPV – negative predictive value

* Validation thresholds chosen to keep the number of false negative the same as the absolute neutrophil count < 0.5x10^9^/L model (47/2237)

**Appendix 6: Twenty Most Important Features from Final GBM Model Fitted on Training Plus Validation Set**

| **Feature** | **Scale** |
| --- | --- |
| ANC 0-<24 Hours | 100.0 |
| Number Platelet Transfusions (7 Days) | 74.2 |
| Mean WBC (7 Days) | 56.8 |
| WBC 0-<24 Hours | 52.5 |
| Minimum ANC (7 Days) | 34.3 |
| Neutropenia 24-<48 Hours | 33.9 |
| Antifungal Two Days Prior | 28.5 |
| ANC 24-<48 Hours | 26.7 |
| Number Previous Positive Blood Cultures (365 Days) | 26.0 |
| ICCC: Acute myelogenous leukemias | 24.4 |
| Sodium 0-<24 Hours | 24.3 |
| Minimum WBC (7 Days) | 22.3 |
| BUN 24-<48 Hours | 16.7 |
| Sodium 24-<48 Hours | 14.3 |
| Minimum Sodium (7 Days) | 14.0 |
| Patient Age | 13.9 |
| Antifungal One Days Prior | 11.5 |
| Number Pathology Specimens (28 Days) | 11.4 |
| ALT 0-<24 Hours | 10.4 |
| WBC 24-<48 Hours | 9.8 |

Abbreviations: ANC – absolute neutrophil count; WBC – white blood cell count; BUN – blood urea nitrogen; ALT- alanine aminotransferase; ICCC – International Classification of Childhood Cancer
